# Efficacy testing of the DSM-5 Cultural Formulation Interview for patients in vocational rehabilitation in Norway

**DOI:** 10.3389/fpsyt.2025.1546150

**Published:** 2026-02-23

**Authors:** Sigrid Helene Kjørven Haug, Valerie DeMarinis

**Affiliations:** 1 Research Center for Existential Health, Innlandet Hospital Trust, Brumunddal, Norway; 2 Faculty of Social and Health Sciences, University of Inland, Elverum, Norway; 3 Department of Public Health and Clinical Medicine, Umeå University, Umeå, Sweden

**Keywords:** Work-related muscoloskeletal disorders, chronic pain, person-centered approach, vocational rehabilitation, cultural formulation interview (CFI), culture, existential dimension of health

## Abstract

**Introduction:**

Patients with work-related musculoskeletal disorders risk becoming outsiders of society due to the complexity of their health and life situations. Chronic pain is often predominant, and the comorbidity rate is high, with anxiety and depression as the most common disorders. Due to the high prevalence and multidimensional character of chronic pain, there has been a call for person-centered and comprehensive approaches that include cultural aspects and an existential dimension. The DSM-5 Cultural Formulation Interview (CFI) is person-centered and was developed to explore all patients’ perceptions of illness, health and support in relation to their life contexts. The study forms part of a larger mix-methods project testing the efficacy of the core CFI (16 questions) in various Norwegian clinical contexts. The objective was to efficacy test the core CFI with patients in vocational rehabilitation treatment.

**Methods:**

The design was inspired by field trials for efficacy testing of the core CFI in the United States. In Norway, the efficacy design went beyond conducting the core CFI in order to follow the use of the CFI information throughout the treatment process. This was referred to as the CFI process. Six consecutive patients were interviewed at three stages: the core CFI on day 1 of treatment, T2 interviews 5-7 days later, and T3 interviews at completion of treatment. Deductive content analysis, following Elo and Kyngäs, was used. Fidelity analysis for conducting the core CFI evidenced high scores.

**Results:**

The main results were: 1) T1-core CFI: Eliciting complex and broad understandings of pain-related problems affecting daily life, 2) T2: The core CFI as a holistic experience, facilitating reflections and hope for the treatment, and 3) T3: The treatment as a significant learning arena with the core CFI as a reflexive basis for treatment processes and expectations.

**Discussion:**

Patients found the CFI process to be complex, positive, and holistic, leading to reflections and expectations for their treatment. This was evidenced further by results from the Debriefing Instrument for Patients (DIP). Future clinical implementation of the core CFI should build on a person-centered foundation, incorporating accountable integration of patients’ treatment expectations and illness/health narrative information.

## Introduction

Musculoskeletal disorders (MSDs) are the primary cause of sickness absence in all member states of the European Union (1). In these patients, chronic pain is a predominant symptom, irrespective of age and geographical location (2). The rate of comorbidity is high, with anxiety and depression being the most common disorders (1). If MSDs are work-related, patients often face complex challenges in both health and work (3–6)^1^,^2^. For instance, extended sickness absence and disability are more prevalent among individuals with lower levels of education compared to those with higher levels (4, 6). Hence, these patients face a significant risk of becoming outsiders in society.

Given the advancements in understanding the interpersonal and multidimensional character of chronic pain (7), there have been recommendations to update the definition of chronic pain by the International Association for the Study of Pain (8, 9). Consequently, the following definition has been proposed: “Pain is a mutually recognizable somatic experience that reflects a person’s apprehension of threat to their bodily or existential integrity” (10). Furthermore, there has been a call to reconsider the approach to chronic pain. This is due to the high rate of opioid prescriptions and the significant personal and social consequences of living with chronic pain (11). The call includes efforts to strengthen person-centered approaches that emphasize a strong therapeutic alliance and shared decision-making (11, 12). Over the past 25 years, research on person-centered care in rehabilitation steadily has increased, highlighting its relevance in the field (13, 14). Interventions that align with individual expectations of care, facilitated through shared decision-making processes, are seen as crucial for implementing person-centered care (13).

### Person-centered approaches

Various holistic models have been proposed to enhance person-centered approaches for patients with chronic pain (15). The most important of these is the biopsychosocial model developed by Engel (16). This model forms the basis of the *International Classification of Functioning, Disability and Health* of the World Health Organization (17, 18)^3^. Consequently, it has been increasingly influential in general rehabilitation (18), particularly in chronic pain management (19). However, the model has been criticized for its underlying biomedical concepts, which often lead to a lack of attention to psychosocial and cultural factors (9, 19, 20). Moreover, the model lacks an existential dimension, which would focus on patients’ suffering and how it threatens meaning in life (21–23). As a result, there has been a call to incorporate new, flexible, and more comprehensive approaches (20, 21).

One such approach is the sociopsychobiological model proposed by Carr and Bradshaw (9, 24). This model includes the above definition of pain, emphasizing that pain is developed and shaped within social contexts. This suggests a focus on the patient’s social and vocational functioning from a health promotion perspective, identifying health factors and resilience (24). A state-of-the-art synthesis on pain psychology in the 21^st^ century highlights that pain management is influenced by the surrounding environment, including social and vocational contexts, and interactions with healthcare professionals in clinical contexts (25). Moreover, it emphasizes the impact of these contexts on individual factors. In the *International Classification of Functioning, Disability and Health* by the World Health Organization, environmental factors are cited as one of the major innovations in the classification, defined as the factors that “make up the physical, social and attitudinal environment in which people live and conduct their lives” (26)^4^. However, culture has proven difficult to operationalize and define in pain research (27).

### Cultural model and the DSM-5 Cultural Formulation Interview

The present study has drawn on a holistic and comprehensive cultural model developed in medical anthropology by Kleinman (28), augmenting this framework with an existential dimension. Kleinman’s work has impacted cultural psychiatry and the definition of culture in the American Diagnostic and Statistical Manual of Mental Disorders, Fifth Edition, Text Revision (DSM-5-TR) (29–31):

Culture refers to systems of knowledge, concepts, values, norms, and practices that are learned and transmitted across generations. Culture includes language, religion and spirituality, family structures, life-cycle stages, ceremonial rituals, customs, and ways of understanding health and illness, as well as moral, political, economic, and legal systems (31).

In this definition, cultural systems are considered dynamic and open. In each person multiple systems are often activated simultaneously, implying daily processes of making sense of experiences and dealing with identity combinations (31). Culture is operationalized broadly as knowledge systems, concepts, values, norms and practices are learned and transmitted, informing human experience and not in the least experiences of health, illness, and the delicate balance between the interplay of individual and society. An individual’s experience of culture involves continuous engagement with their communities and the ways in which these communities understand and navigate factors such as occupation, age, and social class (30). This is especially of importance in social welfare societies such as Norway, where the political goal of enabling as many people as possible to enter the workforce is strongly emphasized as part of the role of contributing to the effective mission of the welfare state (32). Patients with musculoskeletal disorders may experience several barriers in reaching this goal due to the complexity in their life and health situation, often including low socioeconomic status (3–5, 33).

Kleinman’s cultural model includes five dimensions: “biological-physical, psychological, social, ecological, and symbolic” (34). This model has been elaborated further by DeMarinis and applied to healthcare research in Scandinavia by DeMarinis and colleagues (23, 35–42). As all people have an existential dimension, the term “existential” has replaced the term “symbolic” due to the importance of using a concept that is culturally sensitive and includes a wide range of existential meaning-making forms of expression. A culturally appropriate way of understanding the concept of an existential dimension in clinical contexts is as a dynamic, applied, and functional framework that concerns the core of what is most demanding, meaningful, and valuable in daily life for the person (38, 40).

This cultural model has informed the development of the DSM-5 Cultural Formulation Interview (CFI)^5^ (31, 43–45). In this study, we used the 16-item questionnaire (core CFI). The core CFI is based on a person-centered perspective, focusing primarily on the patient’s voice (30). It is divided into four cultural domains: 1) Cultural Definition of the Problem (questions 1-3), 2) Cultural Perceptions of Cause, Context, and Support (questions 4-10), 3) Cultural Factors Affecting Self-coping and Past Help Seeking (questions 11-13), and 4) Cultural Factors Affecting Current Help Seeking (31, 43). The interview offers patients an opportunity to share their illness experiences, including the severity, causes, and progression of their symptoms, available resources, and any social or cultural barriers to treatment that may impact communication and treatment expectations (44, 46). In everyday life, people often draw on multiple sources, depending on the local context and their interactions with different people, such as family, friends, and healthcare professionals (42).

### CFI research

Most CFI studies have been conducted in adult migrant populations in mental healthcare contexts, where the core CFI has been found to be clinically useful, acceptable, and feasible for both patients and clinicians (45, 47, 48). Central findings show improvements in rapport between patients and clinicians, more emphasis on the patient’s voice in treatment planning and care, and valuable contributions to treatment planning and diagnostics (49, 50). The CFI is meant to be used with all patients, irrespective of ethnicity or belonging to majority or minority populations. CFI studies on majority populations is an understudied area, which is now beginning to be addressed (51, 52). A Swedish study on native-speaking patients revealed that the core CFI is a useful, person-centered instrument, particularly for exploring social factors and explanatory models of distress (51). In a Norwegian study in mental healthcare for adolescents, the findings show that adolescents from the majority culture experience the core CFI as a narrative tool to enhance understanding of their needs in treatment (52). The core CFI has been recommended for use in rehabilitation planning to explore the impact of cultural and environmental factors (53), and in vocational rehabilitation to enhance cultural sensitivity (54). However, research in this area is lacking. This study was the first to test the core CFI in a Norwegian vocational rehabilitation center for patients with complex work-related musculoskeletal disorders and possibly mild mental health problems. In addition, it was seen as a contribution to CFI research on majority populations.

### Vocational rehabilitation in Norway

Norway is divided into four health regions. According to the Norwegian Directorate of Health, each region “shall ensure that the population receives social, psychosocial, and medical habilitation and rehabilitation in the specialist health service in accordance with their needs” (55). Referrals for treatment from general practitioners or hospitals are coordinated within each region. Vocational rehabilitation in specialist health services takes place in private outpatient and inpatient facilities. These facilities are financially supported by the state, implying that treatment is free of charge for patients (56)^6^. Patients with musculoskeletal disorders and mild mental health problems are the largest group in vocational rehabilitation (57). According to the Norwegian Council for Musculoskeletal Health, musculoskeletal disorders account for 40% of sick leave, and 32% of disability pension (58)^7^. Thus, musculoskeletal disorders are considered as a major public health challenge.

Internationally, there is no consensus on a common definition of vocational rehabilitation (59). The Norwegian Competence Center for Vocational Rehabilitation uses the following definition for specialist health services:

A time-limited, planned process with clear goals and means and participation in working life as a defined main goal. Several actors collaborate to provide the necessary assistance for the participant’s own efforts to achieve the best possible functional and coping skills, independence and participation in working life (59).

Vocational rehabilitation has a unique position in the healthcare system as it operates independently of diagnoses, focusing primarily on the overarching goal of returning to work (59) ^8^. This is based on the knowledge that work provides structure in everyday life, content and meaning in life, social belonging, and financial security. Additionally, work has positive effects on physical and mental health (6, 60)^9^. Strategies in vocational rehabilitation involve interdisciplinary approaches and multilevel collaborations within the health and social care system. As stated in the legal regulations on rehabilitation of the Norwegian Directorate of Health (61), healthcare should be delivered from a patient perspective and in a meaningful context for the patient. The latest white paper on healthcare competence from the government underscores patient participation as a fundamental principle (62). The principle implies that patients have the right to contribute to and be involved in their treatment and care. A similar focus of letting patients be involved and active in their care is also underlined by the National Competence Center for Vocational Rehabilitation in specialized healthcare contexts (59).

### Study objective

The study objective was to test the efficacy of the DSM-5 core Cultural Formulation Interview for patients in vocational rehabilitation treatment in Norway.

## Materials and methods

### Mix-methods approach and research paradigm

The study forms part of a broader mix-methods project to test the efficacy of the core CFI in various clinical contexts in Norway. Initiated before the COVID-19 pandemic, the larger project is still ongoing. Efficacy is operationalized by feasibility, acceptability, and clinical utility for patients and clinicians in their clinical context, aligning with the approach to efficacy used in the field studies testing the core CFI for the DSM-5 (45, 49). The structured methodological format includes documentation and analysis of different stages in the research process such as: CFI training with clinicians, administration of the CFI by trained clinicians, a fidelity assessment of clinicians’ administration of the core CFI, interviews with patients and clinicians, individual semi-structured debriefing interviews with patients and with clinicians involved in the CFI, and focus group meetings with the larger clinical team and with administrators of the clinic. The format has been further adapted for the project by the second author. This adaptation includes cultural analysis of the clinical sites, and separate efficacy evaluations by clinicians and patients at different stages of the treatment process. More specifically, this involves individual, semi-structured interviews with clinicians at different stages of the research process, and individual semi-structured interviews with patients before completion of treatment. In this way, the core CFI (T1- core CFI) was tested for efficacy at several points in time (T2, T3) during the treatment process. This is described as the CFI process in the project.

Given the research question in the present study, only the efficacy evaluations provided by the patients were included in the analysis to enhance trustworthiness, a fidelity assessment of clinician adherence and competence in conducting the core CFI was utilized (63). A cultural-organizational analysis of the clinical site was conducted to provide background information that would facilitate the following: a) an understanding of the organization’s approaches to person-centered care and culture as stated on its website and related information texts; b) separate meetings with management and clinical staff to assess their understandings of the pilot project; c) observation of the facility and activities in progress; and d) a discussion among clinical staff to ascertain how integrated care and a person-centered focus were operationalized.

**Figure 1:**
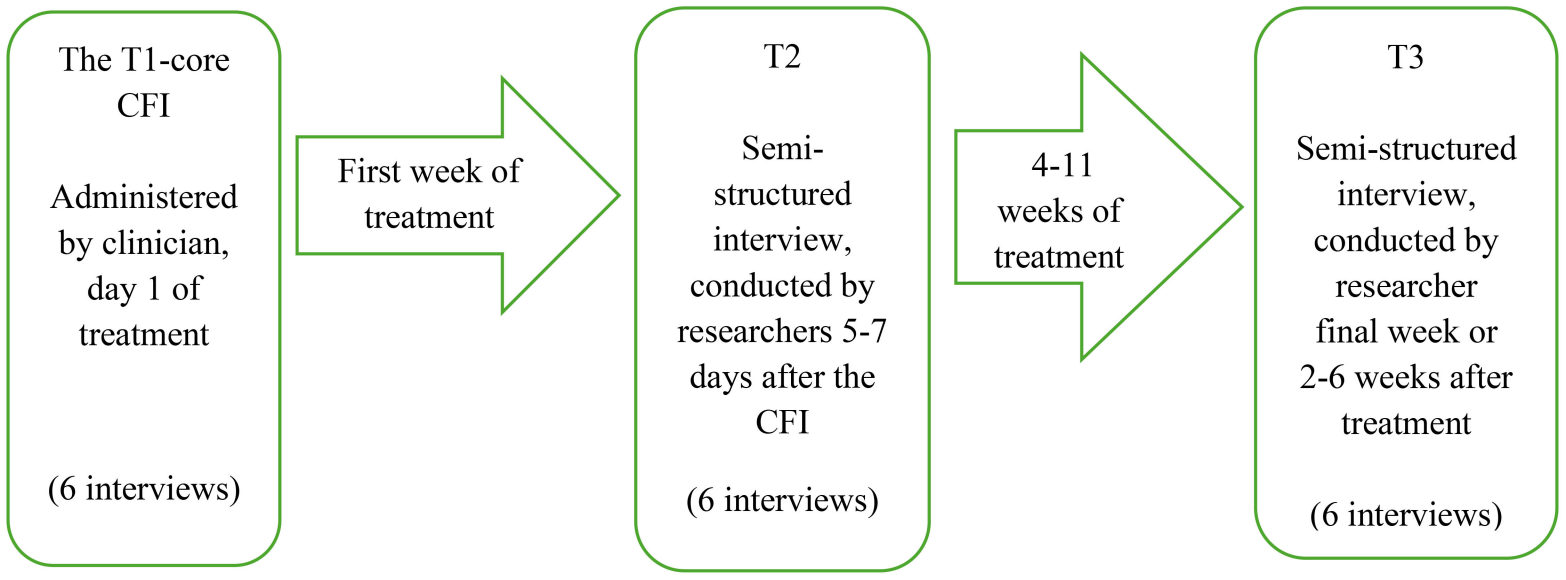
Efficacy evaluation process, patient interviews.

The efficacy evaluation by patients, following the core CFI at TI (T1-core CFI), entailed semi-structured interviews with six consecutive patients at two distinct stages (T2, T3) in their treatment, as depicted in **Figure 1**. The T1-core CFI was conducted by trained physiotherapists on day 1 of treatment. These were followed by the T2 and T3 efficacy interviews, both of which were semi-structured and conducted by the researchers. T2 took place 5–7 days after the CFI. The treatment period ranged from 4–11 weeks, and the T3 interviews were conducted upon completion of treatment or 2–6 weeks thereafter. This gave a total of 18 patient interviews for analysis. Deductive content analysis by Elo and Kyngäs was employed as the analytic strategy (64) (**Figure 1**).

### Researcher characteristics and reflexivity

The authors have expertise in mental health, clinical care, and clinical psychology. The primary responsibility for the larger multi-context project, which aims to implement the CFI in various clinical contexts in Norway, lies with the second author. The first author is jointly responsible and manages the project on a day-to-day basis.

### Context

The vocational rehabilitation facility is situated in a wooded area of Norway. In accordance with the above definition of vocational rehabilitation (59), the primary objective is to enable patients to have an optimal foundation for continuing or returning to work. Patients come from all parts of the country. The selection process is based on a comprehensive strategy aimed at addressing social disparities. This includes patients who are potentially at risk of becoming marginalized in their working life. The approach is interdisciplinary, team-based, and person-centered. Each team is composed of a doctor, vocational therapist, rehabilitation consultant, physiotherapist, sports teacher, and work instructor. The person-centered approach is grounded in certain values and ethics, where every patient is regarded as equal, unique, and capable of contributing to the rehabilitation process. The facility is part of a network for knowledge development operated by the National Competence Center for Vocational Rehabilitation (3). A key partner is the Norwegian Labor and Welfare Administration (NAV), responsible for 1/3 of the state budget (65)^10^. NAV provides financial support for people who are unemployed or on sick leave.

### Sampling strategy

The sample was composed of both patients and clinicians, recruited in partnership with the director and head physician. The clinicians, who were members of the project group, included the head physician, a sports teacher, and two physiotherapists. The physiotherapists conducted the core CFI with patients and were part of each of the two teams at the facility. This ensured that the CFI information was shared with the respective teams.

The CFI study in this clinic followed the methodological format of the larger project: testing the efficacy of the core CFI that included following the CFI process throughout the treatment process for each patient in the different clinical contexts. In each clinical context six consecutive patients fulfilling the context-specific inclusion criteria and formally consenting to being in the study were included. Therefore, six consecutive patients were included in this study. The head physician considered every patient in the rehabilitation facility to be suitable for participation in the study. This assessment was based on treatment referrals to the facility, which contained extensive information about prior treatment and assessments. Therefore, the inclusion criteria here were identical to the admission criteria: patients aged 18–67 years with musculoskeletal problems, possibly a mild mental health problem, a difficult or uncertain employment situation, and usually financially supported by NAV. The exclusion criteria were: an ongoing crisis or traumatic events, psychosis, severe depression, severe suicide risk, and ongoing severe substance abuse.

Recruitment took place in conjunction with the weekly admission of patients. In the introductory meeting with the director, the first author provided information and requested participation. Most patients responded positively on the three occasions recruitment took place, and one person of each gender was recruited each time.

### Data collection methods and instruments

Three types of semi-structured interviews were conducted in an appropriate room at the rehabilitation facility, see overview in **Table 1**. The two physiotherapists in the project group conducted the core CFI following a training course. The duration of these six interviews ranged from 17 to 68 minutes. The first author conducted the T2 and T3 interviews, while the second author participated in the first two T2 interviews and gave feedback on the other interviews. The duration of the T2 interviews ranged from 12 to 30 minutes, and the T3 interviews ranged from 10.5 to 39 minutes. The transcripts from the T1-core CFI were returned to the participants in the T2 interviews for feedback. Also, in the T3 interviews, the transcripts were available for feedback and reflections (**Table 1**).

**Table 1:** Characteristics of the sample and the interviews; T1-core CFI, T2, and T3.

Characteristics: patient, man, female/age	M/40	F/55	F/21	M/25	F/53	M/51
Cultural background, Norwegian, Immigrant	N	N	N	N	N	I
The length of stay in weeks	11	8	4	6	10, 5	4
**T1-core CFI:** Time during admission	Day 1	Day 1	Day 1	Day 1	Day 1	Day 1
Conducted by two physiotherapists	1	1	2	2	2	1
Duration (mins)	1,08	56, 21	37,55	17,11	31,27	52,16
**T2 interviews:** Time after the T1-core CFI	5 days	5 days	7 days	7 days	5 days	5 days
Conducted by 1. and 2. author	1/2	1/2	1	1	1	1
Duration (mins) of T2	24,48	22,43	24,20	12.09	21,37	30,12
**T3 interviews:** Time right before/after discharge	2 weeks before	2–3 days before	6 weeks after	1 week before	2–3 days before	1 week after
Conducted by 1. author	1	1	1	1	1	1
Duration (mins)	20, 28	30,22	39,05	10, 32	19,09	26,44

The interviews; T1-core CFI, T2, and T3 are marked in bold.

The CFI training course consisted of three elements: 1) Preparatory work, 2) One-day training session with behavioral simulations, and 3) Ongoing consultation and feedback. The procedure was inspired by Aggarwal and colleagues (66), as used in the DSM-5 field trials (47). The overall goal was to conduct the core CFI in a person-centered manner.

1. In the preparatory work, the physiotherapists tested the core CFI on themselves, watched a video role-play of the core CFI, and prepared typical and complex cases for the behavioral simulation.
2. In the one-day training session, the entire project group took part. The two physiotherapists participated in the behavioral simulations as both patient and interviewer. This was followed by subsequent discussion of each of the 16 questions, resulting in small adjustments to the core CFI for the patient group, see below. In addition, inclusion and exclusion criteria were established.
3. Ongoing consultation and feedback referred to being available for questions and providing feedback on the CFI process with the six participants.

The core CFI adjustments were applied to questions 3, 8, and 11, see “**Supplementary Material 1**, DSM-5 Cultural Formulation Interview”. The adjustments primarily concerned the addition of sub-questions. They are presented in relation to the primary core CFI questions below:

• Question 3: What troubles you most about your problem?

Here “physically and mentally” was added at the end of the question to specify the two domains.

The following sub-question was added: What are the biggest consequences of these struggles for your daily life? This question was viewed as crucial in treatment planning and for coordination with other services after the treatment period.

• Question 8: For you, what are the most important aspects of your background or identity?

Two sub-questions were included to delve into background resources and problems in detail: What do you perceive as your resources? What do you perceive as your problems?

• Question 11: Sometimes people have various ways of dealing with problems like [PROBLEM]. What have you done on your own to cope with your [PROBLEM]?

Two questions were added to explore coping strategies related to pain, daily life, and work: What have you done yourself to manage your pain? What have you done yourself to manage your everyday life? What have you done on your own to cope with your work or your work relation? Additionally, the question: What gives you strength and courage to move on? was added to question 11 to explore existential information and motivational aspects in depth.

The T2 interview utilized and expanded upon questions from the “Semi-structured, Debriefing Interview Questions on Feasibility, Acceptability, and Clinical Utility”, by Aggarwal and colleagues (48), see “**Supplementary Material 2**, T2 and T3 semi-structured interviews for patients”. T2 concerned patients’ reflections and experiences with the core CFI, and their rapport with the clinician who carried out the interview. Additionally, there were questions about the core CFI experience itself. Among these were questions drawn from the Debriefing Instrument for Patients (DIP) asked orally as part of the interview (67), see **Table 2**, "Patients experience with the CFI (drawing from the DIP instrument)". The questions from the DIP instrument, used as a paper and pen instrument in other CFI research in clinical mental health contexts, were in this study incorporated as oral questions included in the T2 interview. The scoring system was simplified to facilitate the process. The reason for using oral questions included: first time use of the core CFI in a somatic healthcare context; wanting to obtain as much information as possible regarding the core CFI in this context; and, in consultation with the clinical staff noting that oral questions might be more comfortable for the patients. The responses in the instrument range from 0 through 4, increasing in agreement. There is also an option for the response, don’t know (**Table 2**).

**Table 2:** Patients’ experience with the CFI (drawing from the DIP instrument).

Question items	Average response (range)
Helped me explain my main concerns	3.5 (1-4)
Helped me communicate important aspects of my background/childhood, such as religious faith and/or culture	3.7 (3-4)
Helped me understand how my background/childhood and current situation affect my problem right now	2.5 (1-4)
Helped me explain what kind of help I would like	4
Encouraged me to share important information that I might not have mentioned otherwise	3.7 (3-4)
Questions were easy to understand	3.5 (1-4)
Took more time to share my perspective than I wanted	3.5 (1-4) Reverse scored question-Did not take more time than I wanted.
Overall perception of questions	3.7 (3-4)

Response options in question agreement: 0 Not at all; 1 Not very much; 3 Pretty much; 4 Totally, OR (Don’t know)

Notations:

-In general, the patient experiences are very positive concerning these aspects of the CFI. All the patients found that the CFI questions helped them explain the kind of help needed, and emphasized the need to have a voice and be treated with respect. Almost all patients provided concrete examples during the CFI of feeling disrespected and without a voice in relation to prior treatment experiences.

-It is not surprising that the lowest mean score related to background/childhood and current experiences in relation to understanding of their problems now. In this clinical context such background and childhood questions are not areas expected to be covered in a rehabilitation context. Many expressed at first surprise at being asked these questions. However, half of the patients noted in the T2 interview that being asked these questions helped them to get a deeper understanding of their situation.

For four of the six, the questions related to their social networks were difficult ones, bringing to their attention how isolated they felt dealing with their current situation.

-In terms of overall perceptions of the questions, though all found some of the questions challenging, five patients found the questions important. One person was not sure if some of the questions were relevant for the current situation. However, all patients expressed appreciation for the interview and for the sense of trust in the process at the clinic. All patients also expressed a desire for the clinical staff to make use of their information from the CFI in the treatment process and time at the clinic.

Feedback from one of the first patients included regarding support and stresses related to work led to additional sub-questions about working life and NAV in questions 6, 7, and 11 in the core CFI, see **Supplementary Material 1**, DSM-5 Cultural Formulation Interview (43). The sub-questions are presented in relation to the primary core CFI questions below:

• Question 6: Are there any kinds of support that make your [PROBLEM] better, such as support from family, friends, or others?

Two sub-questions were included to explore work and support in managing the loss of work capability: Are there any kinds of support that make your [PROBLEM] better in relation to your work? Are there any kinds of support that help you in dealing with your gradual loss of work capability?

• Question 7: Are there any kinds of stresses that make your [PROBLEM] worse, such as difficulties with money, or family problems?

Three sub-questions were included to explore stresses related to work, gradual loss of work capability, and the relation to NAV: Are there any kinds of stress that make your [PROBLEM] worse in relation to your work? How have you experienced the stress connected to your gradual loss of work capability? If you have had contact with NAV, how have you experienced it?

• Question 11: Sometimes people have various ways of dealing with problems like [PROBLEM]. What have you done on your own to cope with your [PROBLEM]?

One sub-question was added to explore self-coping with work: What have you done on your own to cope with your work or your employment situation?

The questions in the T3 interview were related to how the core CFI had affected the treatment, and overall reflections connected to the importance of the treatment, communication with clinicians, and patient involvement during the treatment period, see “**Supplementary Material 2**, T2 and T3 semi-structured interviews for patients”. The questions were developed by the first and second author.

### Units of the study

The six participants were three men and three women, aged 21-55. Five of the six had an ethnic majority background. One had a minority background but had been living in Norway for 25 years. Four participants were on long-term sick leave, while two worked part time (40%). In addition to work-related musculoskeletal disorders, chronic pain was prevalent. All the participants reported mental health problems. Their relationship to their last workplace varied from non-existent, interrupted and demanding, to supportive. All had a background in manual work, ranging from 2–30 years. Four lived in rural areas and two in cities. Their education level ranged from some years in primary school (1), completion of secondary school (2) and high school (1), to 1–2 years of higher education (2).

### Data processing

The interviews were audio-recorded and transcribed verbatim by the first author. Audio files and anonymized transcripts were stored in a secure research database as directed by the Privacy Protection Department of Innlandet Hospital Trust. Data storage was regulated by the General Data Protection Regulation for EU and EEA member states. Only the two authors had access to the data files. To structure the data, the qualitative data analysis program QSR NVivo (v.14) was used. This program is administered by the Research Department of Innlandet Hospital Trust, and only the two authors had access. During the data analysis, excerpts were checked and de-identified when necessary.

### Data analysis

The selected analytical strategy was deductive content analysis by Elo and Kyngäs (64). More specifically, an unconstrained matrix of analysis was selected as this allowed for a flexible approach in terms of developing categories within the selected core CFI framework. The analysis consisted of three steps: 1) Developing an analysis matrix; 2) Gathering data by content following the principles of inductive content analysis: grouping, categorization, and abstraction, and 3) Developing a model, conceptual system, conceptual map or categories (64). Both authors were involved fully in the three data analysis steps. Any coding disagreements were resolved through further joint analysis until resolution was reached.

1. To develop the unconstrained analytic matrix, QSR NVivo (v.14) was used. This program is widely applied in qualitative CFI research to structure and code the data (48, 51, 68). The unconstrained analytic matrix adhered to the methodology format in the study, see **Figure 1**. The CFI data were divided in accordance with the structure of the core CFI with the four domains. With regard to the T2 and T3 interviews, the entire dataset was included for analysis.
2. Gathering data by content: The grouping continued in NVivo, involving identification of meaning units, which were coded and placed in a hierarchy with newly created main categories and subcategories. During the categorization, the number of categories was reduced by merging those that were similar and moving subcategories that differed to other categories. The abstraction involved developing the main categories further, formulating them in descriptions that were content-specific. This was done manually.
3. Developing conceptual categories: The abstraction process was repeated until the content of the categories was distinctive and the number manageable, leading to the development of conceptual categories. These are presented as main themes and subthemes in **Table 1**.

### Techniques to enhance trustworthiness

To assess clinician adherence and competence in conducting the CFI with patients, clinician fidelity was rated (63), see “**Supplementary Material 3**, Cultural Formulation Interview – Fidelity Instrument (CFI-FI)” (63). This included rating of seven items in the CFI transcripts: Empathy, Patient-centeredness, Clarification, Word matching, Illness narration, Drift, and Order. The two authors rated the transcripts independently, giving a score from 0 to 10. Consensus was reached after discussion. The range varied from 5 to 10 and the mean for all the seven items was 8.3. Drift and Patient centeredness had the highest scores at 10 and 9.7 respectively. In relation to Drift, the clinicians paid attention to CFI topics without combining elements from standard examinations. Patient-centeredness was based on the CFI introductory text stating: “There are no right or wrong answers” (43), implying not correcting or arguing with the patient (63). Word matching had the lowest score with a mean of 6.5. This involved variation in applying the patient’s preferred illness term whenever a question included the term [PROBLEM]. The most common phrases in use were your troubles, your problems, and your situation. The item Clarification had the next lowest score with a mean of 6.8, suggesting that the clinicians could have asked further questions to understand unclear patient responses (63). The lack of clarification mainly concerned mental health problems and symptoms.

### Ethical considerations

The study was conducted in accordance with the ethical standards of the World Medical Association Declaration of Helsinki, and the ICMJE Recommendations for the Protection of Research Participants. The study was approved by the South-Eastern Norway Regional Ethical Committee for Medical and Health Research Ethics (reference number 2016/1703-1) and the Privacy Protection Department of Innlandet Hospital Trust, Norway (reference number 108576). Written informed consent to conduct and publish the study was obtained from patients and clinicians. No participants withdrew after inclusion.

## Results

The results are structured in accordance with the deductive methodological format in the present study as illustrated in **Figure 1**: the T1-core CFI, and the T2 and T3 interviews. The results are presented in **Table 3**, showing main themes and subthemes from each of the three types of interviews. The main theme from T1-core CFI was “Eliciting complex and broad understandings of pain-related problems affecting daily life.” The main theme from the T2 interviews was “The core CFI as a holistic experience, facilitating reflections and hope for the treatment.” Finally, the main theme from the T3 interviews was “The treatment as a significant learning arena with the core CFI as a reflexive basis for treatment processes and expectations.” Due to the sensitivity of the information and to protect anonymity, the quotations are not marked by participant number (**Table 3**).

**Table 3:** Thematic overview of main themes and subthemes.

1. T1-core CFI	2. T2 interview	3. T3 interview
Main theme: Eliciting complex and broad understandings of pain- related problems affecting daily life.	Main theme: The CFI as a holistic experience, facilitating reflections and hope for the treatment.	Main theme: The treatment as a significant learning arena with the CFI as a reflexive basis for treatment processes and expectations.
Subtheme 1: Diversity in pain-related symptoms and background.	Subtheme 1: To be given the opportunity to tell the complete story.	Subtheme 1: Gaining insight into targeted training and recognizing physical limitations.
Subtheme 2: Pain as intertwined with mental health reactions.	Subtheme 2: To be given the opportunity to share expectations and hope for the treatment.	Subtheme 2: The CFI as a facilitator of treatment processes.
Subtheme 3: Pain as invisible and suppressed in social life and work.		Subtheme 3: Satisfaction and disappointment with the fulfillment of treatment expectations.
Subtheme 4: Pain as a threat to life’s foundation.		

### 1. The T1-core CFI information

**Main theme:** Eliciting complex and broad understandings of pain-related problems affecting daily life.

**Subtheme 1:** Diversity in pain-related symptoms and background

The participants understood the complexity involved in their pain problems in relation to their diverse symptoms and backgrounds, the latter ranging from child labor and childhood trauma to burnout reactions and accidents in adulthood. The importance of childhood experience for dealing with complexity as adults was communicated as a form of learning aimed at ensuring a better life for their family and themselves. Simultaneously, it acted as a limiting factor due to enduring health implications.

The burnout reactions were linked to heavy burdens in both private and working life over several years. Exhaustion was highlighted as a dominant symptom, with extensive repercussions in daily life as characterized below:

Being exhausted is the biggest thing now, which means that I can’t do much. I tire easily, and then I become shaky, feel terrible and unwell. So, I’ve got no control over these reactions. I can do things for a few hours, and that works out well, and suddenly I get completely empty and then I must go back home and lie down.

Accidents were expressed as catastrophic, having negative consequences for work and daily life with descriptions such as: “I was totally shattered, and I couldn’t do much afterwards”. Shortly after the accident, the intense and incomprehensible pain sparked fears of life-threatening diseases like cancer. Another response was forced inactivity due to nerve pain.

The participants explained how the pain-related symptoms disrupted their daily lives. Three common aspects emerged. Firstly, the participants described the high levels of pain they felt throughout their bodies, with phrases like: “my body is completely locked”, “it’s like a toothache in my whole body”, “the pain’s been hell”, and “my whole body’s filled with pain”. Other less specific descriptions included “feelings of inflammation and irritation” and “feelings of drowsiness”.

Secondly, the demanding nature of the pain-related problems was associated with a gradual worsening of pain over the years, encapsulated by the phrase “more and more problems in my body over the past years”. This decline had negative impacts on crucial areas of daily life, such as sleep, regular activities, work, social life, and mental health. Limitations in daily life were detailed: “hard to lift up a cup or bend down” and “I get pain if I walk more than a few meters”. Adjusting the level and intensity of activities was felt to be demanding and frustrating. This gradual worsening was attributed to external stress factors such as poor living conditions, harsh working conditions, difficult family relationships, and negative experiences with treatment. Specifically, manual labor involving heavy lifting and harsh working conditions was highlighted as having a strong influence. These working conditions also included long hours, cold indoor temperatures, and a lack of managerial support.

The third common feature was the recognition that the challenging nature of the pain problems suggested that there were still unknown factors to be evaluated. This uncertainty made it challenging to comprehend and manage the pain: “I wish for something magical to happen that could help me, a stroke of luck.”

**Subtheme 2:** Pain as intertwined with mental health reactions.

The participants acknowledged the complexity of the pain-related problems in relation to mental health reactions, viewing them as intertwined. The mental reactions covered a broad range, as illustrated with terms such as psychic pain, anxiety, depression, panic attacks, trauma, stress reaction, despair, frustration, sorrow, loss, bitterness, worries, difficult body image, and loss of control.

The participants elucidated their struggles in understanding and distinguishing between the mental aspects and the pain. Recurring episodes of exhaustion had led to panic attacks, frustration, overwhelming sorrow, and depressive reactions. The difficulty of figuring out how to cope was also expressed: “I can’t breathe properly, and I don’t know what to get rid of first, the pain or what’s inside here”. Traumatic and crisis-related experiences from childhood were seen as causes of pain-related problems, including parental addiction problems, sexual abuse, contentious divorces, child labor, an unsupportive environment, and unspecified crises.

The participants detailed their efforts to function as well and normally as possible in their work and personal lives. This included striving to become more athletic, robust, and active. Managing frustrations when the pain level made them adjust their activities was described as challenging. Similarly, maintaining positivity and living as normal a life as possible was described as a mental struggle: “It’s hard to be positive and work while my body says no all the time and works against me”. In such cases, the prospect of adjusting to a lower activity level was more frightening due to past experiences of depression or a loss of control during inactive periods.

**Subtheme 3:** Pain as invisible and suppressed in social life and work.

The participants conveyed how their pain was often invisible and suppressed in their social and working life, falling into two patterns. The first involved concealing the pain from others to avoid appearing weak or incompetent. Consequently, social interactions were often experienced as challenging and draining, leading to social withdrawal. Low energy level was a dominant symptom, triggering prolonged and difficult-to-resolve conflicts in close relationships. The participants’ social backgrounds in childhood, characterized as being “poor”, “traumatic”, “bad”, “no time to be a child”, or “brought up in an orphanage” influenced this pattern. However, in one instance, the person’s childhood was described as “good”.

The second pattern related to the invisible nature of the pain, which made explaining it to others necessary but demanding: “I was depressed at the time, although I’ve normally been strong in that sense. I almost lost control of myself when people asked how I was doing, I just started to cry”. Typically, the participants either concealed the severity of their pain or shared it confidentially with those closest to them. For some, this had resulted in loss of friendships. In this situation, their family members, such as a partner or parent, played a central role in dealing with the pain.

Loss of work capability, either gradual or abrupt, was described as “more of a burden than the pain problems”, and “a traumatic process”. In the latter case, it was related to an accident, leading to feelings of “bitterness, sadness, and despair”. Support at work was crucial, and without it, managing the pain became an even more challenging task. The struggle of dealing with pain in conjunction with difficult working conditions was illustrated with the metaphor “a vicious circle”:

I’ve been told that nothing can be done about my worn-out back and that I must live with it. What’s more, I’ve been advised to exercise and be active to strengthen my muscles so the pain gets less. But the cold temperatures at work make the pain so bad that I can’t exercise when I’m at my worst. My situation turns into a vicious circle where my body in the end is totally locked. Then I have to go to the chiropractor to sort it out.

The participants pointed out how their mental health was heavily influenced by their work and financial situation. An unpredictable and difficult financial situation led to fear and concerns about the future. This was a key reason why they pushed themselves to work, striving to do their best without complaining. The long-lasting impact of this process was described as follows:

My thoughts are like a roundabout, with all the cars driving into it but none driving out. As a result, I can’t think straight. My thoughts jump in different directions all the time without finding their way out. So, I can’t sleep.

**Subtheme 4:** Pain as a threat to life’s foundation.

The participants understood the complexity of the pain problems in relation to the way it threatened the very foundation of their lives. This foundation was confronted in relation to identity, loss of work capability, and hope for the future. Identity was expressed through key phrases about themselves and their life. These key phrases exhibited two distinct patterns. The first pattern was laden with phrases that the participants used to describe themselves and their life experiences. These included life-span reflections such as “I was born with bad luck”, “I learned from my childhood to always push myself”, and “Is Karma the cause of my pain?”

The second pattern in key phrases revolved around self-characteristics in relation to daily life: “Everybody sees the change as I’m really a toastmaster type of person”, “I hate to appear weak”, and “I don’t get more trouble than I can bear”. The latter phrase described a life attitude fostered and strengthened by sharing it with a close relative.

In one case, identity and body image were felt to be connected, as illustrated in the following dialogue with the clinician (C):

P: For me everything shuts down when I can’t fix the things that come up.

C: What do you mean by shuts down?

P: Then I get very depressed. As I mentioned earlier, I couldn’t even go running. People much older than me are running. And here I am, much younger, just hanging and messing around. My self-esteem, quality of life, and well-being are tied to being athletic and strong. This is my identity. So, it gets shaken in…

C: The foundation…

P: Yes, the foundation, and it’s seriously shaken too.

The ability to “fix things” was linked to work and the confidence derived from being good at it. A question in this connection was “When my body’s stopped working, what do I have left?” The participants considered work, close relationships, and care of animals as most important for providing strength to move on in life. They emphasized the stress of losing work capacity due to the importance of work in shaping their identity, self-worth, and well-being. A common sentiment was: “I really want to work 100% because my work has always been a part of my life”. The fear of being perceived as weak and a failure in society was a prominent concern.

In terms of close relationships, the participants highlighted how the pain problems often resulted in feeling lonely and being a burden. They described their efforts to compensate for this by suppressing their pain, pushing themselves in social activities, and adopting a solution-oriented mindset. Daily care of animals was described as a source of motivation to keep going in life: “The horses are always glad to see me.”

The participants described both worries and hope for the upcoming treatment and the future. Expressions of worry about the treatment were associated with negative experiences of previous treatment, including feeling distrusted, undervalued, misunderstood, or dismissed. Regarding the relationship with clinicians, hope was associated with the opposite feelings, such as being respected and understanding the clinician’s words. Using ergonomics at work was also considered crucial. Worries about the future were centered on work capability, financial stability, social standing, and potential dependency on painkillers. Participants’ expressions of hope for the future included being able to work at full capacity, providing better care for their family, and enhancing their physical condition.

### 2. T2 interviews, 5–7 days after the core CFI

**Main theme:** The core CFI as a holistic experience, facilitating reflections and hope for the treatment

**Subtheme 1:** To be given the opportunity to tell the complete story.

Most participants stated that the core CFI provided an opportunity for them to tell their complete story. The approach of narrating “at length” was described as a novel experience, something that “had never happened before”. Participants found a clear contrast between the questions addressing them as persons or addressing the contexts of their lives, and those that focused solely on their illness and symptoms:

I’ve never been asked questions about who I am as a person. Considering how the pain problems have affected me, it’s been quite traumatic since I’ve never been sick before.

No one has ever gone so deep into things. Generally, conversations have sort of ended before that.

I can’t compare it to anything specific, but I think the interview was very good and refreshing. Finally, someone paid attention to the circumstances around me, not just the disease, and then went on from there. Those are factors that influence how you feel.

The unique nature of the questions was highlighted as a defining feature, prompting reflections on past lifestyles, strategies for better handling of life in the future, and the impact of background information on the treatment, and expectations for it. Question 7 about stresses that make the problems worse, and question 8 about identity and background were emphasized as enlightening. Insights into the interplay between financial circumstances and mental health were highlighted as important in question 7. In one case, the sub-questions about the challenging consequences of losing work capability and the person’s relationship to NAV were suggested, as detailed in the method section. One response criticized the questions about the social network (questions 2, 5 and 15), finding them repetitive and challenging due to a lack of network support in the patient’s situation. This was illustrated with the metaphor “being a lone wolf”.

**Subtheme 2:** To be given the opportunity to share expectations and hope for the treatment.

The participants felt that the core CFI provided them with an opportunity to share their expectations and hopes for the forthcoming treatment. This was tied to three elements: reactions to the core CFI, initial impressions from their first days in the unit, and previous encounters with healthcare professionals in various settings.

The participants’ experiences with the core CFI encompassed both their responses to the questions and their relationship with the clinician conducting the interview. The questions were viewed as non-offensive, friendly, and elaborating, bolstering their hope that help was attainable. The questions spurred reflections on their attitudes towards treatment and their prospects for change, as exemplified in the following quote:

I believe that those questions boosted my motivation for my stay here. I started off somewhat skeptical. But perhaps the questions can help me to be more receptive, to think things over ahead of time, as I might need to rediscover myself, find out who I really am, who I’m aspiring to be.

As for the relationship with the clinicians, the participants appreciated feeling welcomed, cared for, listened to, and believed right from the outset. These factors fostered an expectation that their issues would be handled appropriately. The act of being listened to was described as an opportunity to narrate their full story without being steered in a specific direction. Feeling cared for, in the sense of being “heard and believed” from the start, was crucial and described as a departure from just being pitied. For some participants, treatment expectations and hope were intertwined, including notions about what might be realistically achievable, as illustrated in the following two quotes:

The interview gave me hope that I can get help. It didn’t affect me negatively, and I continue to make an effort to think positively. I understand that they don’t perform miracles here, so I try to manage my expectations.

Perhaps they can work on my background, which would be interesting, but it might also be challenging.

The initial impression from the first days in the unit was predominantly positive, as described in the following statement: “Although I’ve only been here a short time, I genuinely believe this is a very good place. I was a bit skeptical beforehand”. However, one participant shared an example of not being heard, leading to feelings of withdrawal and panic that the treatment would mirror past experiences.

The final element of the interview about previous interactions with clinicians in various healthcare contexts revealed that these interactions were either described as beneficial or challenging for the participants’ rehabilitation process. Helpful experiences included being listened to and allowing despair and sorrow to be expressed. Stories about challenging experiences included limited consultation time, which hindered information exchange and understanding, and perceived arrogance by healthcare professionals. This arrogance was described as a lack of understanding, a controlling demeanor, and tunnel vision in relation to the information the patients presented. Furthermore, examples were given of feeling unheard and dismissed during multiple periods of assessment and treatment. This was contrasted with the experience of the core CFI:

I’ve found that the clinicians sometimes make the problems worse than they are, or one aspect of the situation is blown out of proportion. Instead, they should have focuses on all the elements that affect how I felt. In the core CFI, I could express myself based on the actual situation without being steered by the interviewer in one direction or another. I believe this approach is much better.

### 3. T3 interviews, final two weeks or after completion of treatment

**Main theme:** The treatment as a significant learning arena with the core CFI as a reflexive basis for treatment processes and expectations.

**Subtheme 1:** Gaining insight into targeted training and recognizing physical limitations.

The participants shared a common realization that the treatment offered insights into targeted training and raised awareness of their physical limitations. The atmosphere in the unit and the clinicians’ attitudes were identified as key to gaining these insights, with descriptors such as “positive”, “supportive”, and “respectful”. Moreover, being with other patients was felt to be inspiring and supportive of the benefits of their stay. This included doing activities together, and sharing personal experiences, the latter being highlighted in the following way: “We shared a lot, for instance about physical training, work, and how to solve our problems. All of us are striving to recover fully”.

One insight gained was how physical activity could function as a pain management strategy. Specific goals, such as weight loss, muscle strength improvement, increased body flexibility, and pain reduction were achieved through the physical training sessions. The reduction in pain levels was evident from decreased pain medication intake, which in turn led to fewer side effects throughout the day. Gratitude for the improved muscle strength that resulted from the extended treatment period was expressed in the following way: “I’m very grateful that I could extend my stay. I’m going back to a physically demanding job, and I feel better equipped after I’ve spent some extra time here”. Such insights also led to a change in attitude towards regular training: “The way I look at physical training has changed from seeing it as just exercise to seeing it as something necessary I can do at home”. The participants also highlighted the need for further assessments and examinations due to unresolved questions about their pain.

Awareness of physical limitations was tied to accepting pain-related issues, which included recognizing the need for relaxation. This involved lessening the pressure on oneself, as illustrated in the following dialogue:

I: So you say you’ve accomplished a lot while you were here.

P: I feel that I did what was needed and in the beginning I did a bit too much. I had to reduce my pace because I wanted to make the most of my stay here, to learn everything and perform as well as possible.

The challenge to convey one’s limitations to others was also tackled: “Despite the pain, I’m making a strenuous effort to recover and get back to work. I don’t want to appear weak”. There were instances where the limitations were either misunderstood or too overwhelming to manage during the treatment at the facility. This led to depleted energy and increased pain throughout the treatment period, as illustrated in the quote below:

I lost my physical energy and mental capacity during my stay. It was an intense course of training on an already exhausted and tense body, which might have been too much.

I attempted all the activities, but I couldn’t do them because I can’t bend over and hold something in my hand, and I can’t lift anything. If I do, I get severe headaches, and so I relied a lot on headache medicines and other drugs during my stay.

**Subtheme 2:** The core CFI as a facilitator of treatment processes.

Most participants emphasized the role of the core CFI in facilitating the treatment in two significant ways. First, being welcomed with the core CFI on day 1 of the treatment instilled a sense of trust and safety right from the start. These feelings were reaffirmed several times during the treatment. A similar experience was being listened to without being judged.

Second, the core CFI initiated various reflections about the need for personal changes, such as allocating time for rest, addressing childhood trauma, and identifying personal resources and goals for physical training and future planning. In one case, the recommendation was to find a symbol from nature that could serve as a reminder of these changes. This experience was shared as follows:

P: I was out for a long walk in the countryside, I didn’t look for anything but when I saw the stone, I thought there it is.

I: There it is.

P: There it was, a calming down stone was waiting for me. I feel kind of safe now, much safer than before I came here.

Some participants found it difficult to recall information from the core CFI due to the considerable time gap between the interviews. In one case, the possibility of sharing similar information with their doctor was perceived as more crucial to the rehabilitation process.

**Subtheme 3:** Satisfaction and disappointment with the fulfillment of treatment expectations.

The participants shared mixed feelings of satisfaction and disappointment regarding the fulfillment of their treatment expectations, in relation to what they had shared in the core CFI. The satisfaction primarily related to insights gained through targeted physical training, goal achievement, and awareness of physical limitations as detailed in subtheme 1. This satisfaction was expressed as acceptance of pain and limitations: “I’ve come to accept that I live with pain and limitations. It’s now more about improving my quality of life, listening to my body, not just pushing myself”.

The participants highlighted the positive atmosphere in the unit as key to meeting their treatment expectations: “No matter where you look, people are positive. It must be something they focus on strongly, so they can make us positive too and get everyone involved”.

Moreover, the predictability provided by the schedule and routines was felt to be crucial, as it was difficult to establish similar regularity at home.

However, some participants also expressed disappointment at treatment expectations not being fulfilled. One area was related to question 8 in the core CFI about background and identity, where they contemplated what might have transpired had such issues from the CFI been included as part of the treatment. Another disappointment stemmed from the high intensity of the treatment program, which led to increased pain levels: “Despite this, I made the attempt, but it didn’t work out”.

A final area of disappointment was the feeling that the information from the core CFI was not adequately integrated into the treatment, suggesting that the communication was complex:

I had no more contact with the clinician who interviewed me with the core CFI. There was poor communication between me and them. I genuinely felt that they didn’t use that information for anything, and I was very disappointed. And then, at the final meeting, they said something completely different than what they said before.

In this case the sense of a lack of inclusion of important information shared for the first time in the CFI contributed to a worsening of the patient’s overall situation.

## Discussion

The objective of the study was to test the efficacy of the DSM-5 core Cultural Formulation Interview (core CFI) for patients in vocational rehabilitation treatment in Norway. The results from the three interviews, T1-core CFI, T2, and T3, revealed that the participants experienced the CFI process as follows: T1-core CFI: Eliciting complex and broad understandings of pain-related problems affecting daily life; T2: The core CFI as a holistic experience, facilitating reflections and hope for the treatment; and T3: The treatment as a significant learning arena with the core CFI as a reflexive basis for treatment processes and expectations. This was the first study to test the efficacy of the core CFI in a somatic healthcare setting in Norway. Although the core CFI has been advocated for use in rehabilitation in general (53), and specifically in vocational rehabilitation to enhance cultural sensitivity in healthcare professionals (54), research in this area remains scarce. Furthermore, CFI studies on majority populations have been limited (51, 52), and the study is seen as a contribution to filling this gap.

The study responds to the call for reframing the understanding of chronic pain by strengthening person-centered approaches (11, 12). Additionally, it aligns with the recommended need to employ flexible and comprehensive approaches that include culture and the existential dimension (9, 19–22). The present study drew on a holistic and comprehensive cultural model of health developed in medical anthropology by Kleinman (28) and adapted to Scandinavian contexts by DeMarinis (). It includes five interacting dimensions: “biological-physical, psychological, social, ecological, and existential” (34). Furthermore, the broad understanding of culture from the revised version of the DSM-5 and DSM-5-TR was applied to capture the variety of cultural systems that influence daily processes of making sense of experiences (31, 34, 52). We employed the core CFI, a validated clinical and person-centered tool in mental healthcare (31, 43, 44, 69), which is informed by Kleinman’s cultural model and the DSM-5 definition of culture. The core CFI is the standard cultural assessment process in the DSM-5, designed to amplify the patient’s voice and the patient’s interpretation of the voices of significant others in the patient’s life (30, 31). This approach is in accordance with the service user perspective outlined in the legal regulations on rehabilitation from the Norwegian Directorate of Health (61), inferring that patients have the right to contribute to and be involved in their treatment and care. This is further underlined in the latest white paper on healthcare competence from the Government (62), and the updated document on work and health from the National Competence Center for Vocational Rehabilitation (59).

The overall finding in the study was that the participants perceived the CFI process as complex, positive, and holistic, fostering a reflexive basis for treatment processes and expectations. The complexity was tied to their health and life situations as well as the treatment process. Research on patients with work-related musculoskeletal disorders shows that they face potential marginalization in society due to complex health and life situations (3–6, 33). Such complexities include chronic pain, mild mental health problems, and low education level, often resulting in extended periods of absence from work, difficulties in returning to work or disability pension. These aspects were of concern to the study participants who represent the largest group in vocational rehabilitation in Norway (56). This group also accounts for the highest percentage of sick leave and permanent disability (57).

The treatment process with the core CFI elicited a wide range of participant perspectives on the everyday consequences of living with chronic pain. These included diversity in pain background and symptoms, the interrelation of pain with mental health reactions, the attempt to conceal pain in social life and work environment, and the perception of pain as a threat to life’s foundation. Each of the subthemes encompasses the range of dimensions in Kleinman’s cultural framework^31^, signifying their successful encapsulation of the multifaceted nature of living with chronic pain, as emphasized in chronic pain research (7). Moreover, the T1-core CFI contained a variety of illness explanatory models, based on symptoms, background, progression, and socio-cultural barriers to treatment (44, 46). Identifying these barriers is crucial when conducting the core CFI as they potentially can influence clinical communication and treatment expectations.

Three significant implications of living with chronic pain, that emerged in the T1-core CFI, are presented here. The first centered on the consequences of diminished work capability, primarily concerning an uncertain financial situation. This greatly affected the participants’ mental health, arousing feelings of fear and concern. Further, the participants perceived their overall situation as challenging, both socially and existentially, and this was the main reason for them to persist in their work despite their pain symptoms. This probably reflects the risk they faced of becoming marginalized in society, a notion supported by research on this patient group (3–6, 33). It also highlights the relationship between work and health, revealing the crucial role work plays overall (59) and its positive impact on both physical and mental health (6, 60). The participants viewed mental health problems as interwoven with chronic pain problems, complicating the process of differentiating between them. This was attributed to past trauma and crises. This insight is paramount, as anxiety and depression are the most common co-occurring disorders in patients with work-related musculoskeletal disorders (1).

The second implication involved the participants’ social life, where they hid their pain from others to avoid being perceived as weak or incompetent. Their challenging social backgrounds, characterized by adjectives such as “poor” and “traumatic”, were seen as primary reasons for this concealment of pain. Both the work-related practice of persevering despite the pain, and the hiding of pain from others, can be viewed as social and cultural barriers to treatment that need to be addressed to prevent misunderstandings. The identification of these barriers support previous findings on the usefulness of the core CFI in exploring illness experiences of distress in majority populations (51). The significant personal and social consequences of living with chronic pain are well-documented (9, 11, 24). This understanding is reflected in the new definition of pain by the International Association for the Study of Pain (10), as well as in the sociopsychobiological model proposed by Carr and Bradshaw (9, 24). The definition emphasizes intersubjectivity with respect to pain, as well as involving a social process which enables appropriate responses from others. Threats to bodily integrity, understood as compromising wholeness, are so severe that they are perceived as existential threats (10).

The final implication concerned existential threats, including identity, loss of work capability, and the struggle to balance hope and future worries. The loss of work capacity appeared to be the biggest existential threat, considering the role of work in shaping identity, self-worth, well-being, and hope for the future. The participants expressed a fear of being viewed as weak and a failure in society. Past experiences of misunderstandings and devaluation during treatment were recounted, with the hope for respect and effective communication in the upcoming treatment. Future hopes and concerns also pertained to life post-treatment, primarily related to work capacity, financial stability, and family care. This understanding of the existential dimension stems from Kleinman’s cultural framework and DeMarinis’ definition of the existential: a dynamic, functional, and applied framework, closely tied to the most challenging and meaningful aspects in daily life (38, 40). In Scandinavian healthcare research across various populations (23, 35–40), these areas have been identified as culturally sensitive expressions of existential meaning-making, which become actualized in specific life situations and clinical contexts. This finding aligns with CFI research in Norway on adolescents in mental healthcare, where the existential dimension informs decision-making and daily life management (46). To fully understand the impact of the existential threat posed by losing work capacity, both on an individual and societal level, it is essential to adopt a broad definition of culture, as outlined in the DSM-5-TR (31), which includes political systems as a cultural factor. The political goal of enabling as many people as possible to enter the workforce is strongly emphasized in Norway (32). This goal influences how patients perceive and manage their life and health situations as well as their assessment of their own self-worth to themselves and to society. This highlights the need for these factors to be addressed specifically in vocational rehabilitation to support the patients in the process of returning to work or finding a way of making an active contribution to the welfare state’s function. In this respect Norwgian culture encompasses an existential dimension that always includes the political. Not being able to meet this political goal can be an initial or long-term source of great existential distress for individuals, with both physical and mental health consequences.

The main findings from the T2 patient interviews (about 5–7 days after the T1-core CFI) showed that the core CFI was viewed as a positive and holistic experience, fostering reflections and promoting hope for treatment. This sentiment was linked not only to the relationship with the clinician who conducted the core CFI, but also to the nature of the questions asked. The participants felt acknowledged and valued, and they believed that they were genuinely listened to right from the start. The questions were perceived as person-centered and expansive, sparking thoughts on a variety of areas, such as potential future strategies and the relationship between their financial situation and their mental health. Furthermore, it strengthened their belief that help and support were available. The high scores on the DIP questions provided further evidence of the positive and holistic experience. The clinical acceptability and utility for the participants corresponds with central findings from the CFI field trials (45, 47, 48) and broader CFI research (50, 69), demonstrating that the interview enhances rapport between patient and clinician, while also improving communication.

In both the T1-core CFI and T2 interviews, the participants shared experiences from previous interactions with healthcare professionals. These experiences were a mix of helpful and challenging encounters. Helpful experiences often included instances where they felt heard, and were asked questions similar to those in the core CFI. Challenging experiences largely involved perceived arrogance from healthcare professionals in the form of a lack of understanding and a tunnel vision approach to the information the patients presented. In the T3 patient interviews (at the end of treatment or shortly after), the participants expressed disappointment with unmet expectations during the treatment period. These concerned a mismatch between the high level of pain and the intensity of the treatment program, and communication difficulties and mistrust due to the lack of integration of the core CFI information into the treatment process. These findings underscore the importance of prioritizing person-centered approaches, emphasizing the therapeutic alliance and shared decision-making processes that include patients’ treatment expectations (11, 12). Additionally, the findings highlight the responsibility of the unit to follow up the CFI information to avoid mistrust and treatment interruptions.

The final main finding was that the CFI process created a reflexive basis for treatment processes and expectations, forming a common theme running through the treatment that could be added to, altered, or adjusted. This was a key theme identified in both the T2 and T3 interviews. In the T3 interviews, the participants shared how the clinicians’ attitudes and the positive atmosphere in the unit were important for the beneficial outcome of the treatment. Outcomes included goal achievement such as increased muscle strength and lower pain levels. The use of the T1-core CFI on the first day of treatment had established a feeling of trust and safety right from the start. Further, the participants conveyed how the T1-core CFI sparked thoughts about the necessity for certain changes such as addressing childhood trauma and making use of personal resources for future planning.

The patients in this study came from a wide geographical region in the southeastern part of Norway. The broad and dynamic understanding of culture outlined in the DSM-5-TR (67, 68), which serves as the foundation for the core CFI, functioned well as a framework for capturing the diversity of their backgrounds, living and working conditions, health challenges, and both personal as well as interpersonal resources. This framework helped contextualize their experiences of physiological, psychological, social, and existential strain within a broader cultural perspective. As a result, the findings of this study underscore the value of the core CFI in integrating cultural psychiatry and medical anthropology into clinical practice (69).

A limiting factor at the organizational level was observed. Despite the frequent naming of severe psychological concerns like trauma in the core CFI, the unit did not provide integrated care for such issues.

### Clinical and research implications

The core CFI aligned well with the patient-oriented and person-centered foundation of the rehabilitation facility, where the patient was acknowledged as an equal and competent contributor (62). On this basis, the main purpose of the core CFI, which is to strengthen the patient’s voice in treatment and care, was accomplished (30). A clinical implication derived from this finding is the importance of developing similar person-centered foundations in contexts where the core CFI is to be used. In this way, the interview will have the potential of being fully integrated in patients’ treatment processes. Given that patients with musculoskeletal disorders pose the greatest public health challenge across the member states of the European Union (1, 2), there is a general need for knowledge of the integration of person-centered approaches in vocational rehabilitation (13, 14) and chronic pain management (10–12). More specifically, this also applies to CFI research in vocational rehabilitation (50), as this has been limited. The importance of a person-centered foundation, and its direct operationalization through methods such as the CFI, are highly relevant in healthcare contexts in general, as person-centered approaches are emphasized across various fields (70)

Another clinical implication of the study is the necessity to integrate patients’ treatment expectations in a responsible manner, as disappointment may lead to mistrust during clinical encounters. We suggest that the expectations for care elicited by the core CFI are integrated into shared decision-making processes rooted in a person-centered foundation, as recommended in a systematic review on person-centered rehabilitation care (13).

Kleinman’s cultural framework functioned well in the study, hence we recommend this framework in future research (28). This cultural framework, including the existential dimension (40), addresses the restrictedness of the biopsychosocial model (9, 19–21). For the participants in this study, mainly belonging to the majority population, the core CFI was experienced as a brief intervention, initiating reflections on different levels. Based on findings in the larger CFI project on efficacy for patients and clinicians, implementation research has been initiated. This will add more knowledge to the clinical and research implications of considering the core CFI as a brief intervention.

### Strengths and limitations of the study

As the current study was part of a larger mix-methods project testing the efficacy of the cor CFI for both patients and clinicians in different clinical contexts in Norway, the study followed the same methodological format as in the larger project. The design concerned efficacy testing of the core CFI with six consecutive patients, interviewed at three different points of time. The small sample size is a limitation in this study. However, the total of 18 patient interviews permitted following the CFI process in the treatment process for these six patients at three points in time. This provided rich and nuanced descriptions that offered unique insights into the comprehensive CFI process for the specific patient group. The open data-gathering approach, in which patients were invited to participate in the initial meeting on day 1, worked well as the inclusion criteria matched the admission criteria. Interviewing patients with the core CFI on the first day of treatment aligns with the DSM-5 TR recommendation of timing of the CFI (31). Barriers related to memory and language may have affected the content of some interviews. The time lapse between the T1-core CFI and T3 interviews presented some challenges for the patients in recalling the core CFI and its influence on the treatment process. To mitigate this, they were encouraged to refer to a copy of the core CFI during the T2 and T3 interviews, leading to feedback and deeper reflections in some cases. Additionally, patients shared their feedback on the treatment- and CFI process during the T3 interviews. All this information was incorporated into the study findings. The clinicians in the project group provided feedback on the findings during separate efficacy evaluations and discussions; however, these findings are not included in the present manuscript.

The CFI-FI assessment yielded a relatively high fidelity score with a mean of 8.3 out of 10. This is due to the comprehensive CFI training course, which involved preparatory work, a one-day session with behavioral simulations, and ongoing consultation and feedback. The item “word matching” had the lowest score, emphasizing the need to include this item in future training to ensure that the patient’s preferred illness term is used by the clinician for the term [PROBLEM] in the core CFI. The item “clarification” scored next lowest, mainly due to insufficient exploration of patient-reported mental health problems.

A scoping review on CFI research indicates that the core CFI is typically administered by psychiatrists, with only a few studies involving psychologists and social workers (47). However, in the present study, CFI-trained physiotherapists demonstrated strong clinical skills in administering the CFI. This suggests that instead of restricting the administration of the core CFI to specific professions, an interdisciplinary approach should be adopted to determine who in the clinical team is best suited for the task.

The use of QSR NVivo (v.14) in the deductive content analysis offered several benefits, including its capacity to organize initial codes and their grouping, as well as serving as a foundational database for identifying citations. No disadvantages were observed. Hence, QSR NVivo (v.14) is recommended for use in qualitative CFI research in the future.

The analysis involved close collaboration between the two authors, who oversaw the development of the unconstrained matrix of analysis in NVivo. Further collaboration in the analysis included categorization, and abstraction of categories, leading to final themes and subthemes, thereby enhancing the trustworthiness of the findings.

## Conclusion

This was the first study to apply the core CFI in a somatic healthcare setting in Norway. Furthermore, it is one of the few CFI studies conducted on majority populations. The core CFI was seen as an appropriate instrument due to the person-centered basis in the vocational rehabilitation facility. The patients found the CFI process to be complex, positive, and holistic, leading to reflections and expectations about the treatment. The high scores on the Debriefing Instrument for Patients (DIP) provided further evidence of the positive and holistic experience. The clinical acceptability and utility for the patients corresponds with central findings from the CFI field trials and broader CFI research, demonstrating that the interview enhances rapport between patient and clinician, while also improving communication.

The broad and dynamic understanding of culture outlined in the DSM-5-TR, which serves as the foundation for the core CFI, functioned well as a framework for capturing the diversity of patients’ living and working conditions, health challenges, and resources. This framework helped contextualize their experiences of physiological, psychological, social, and existential strains and resources within a broader cultural perspective. Future implementations of the core CFI should build on a person-centered foundation, incorporating accountable integration of patients’ treatment expectations and illness/health narrative information.

## Data Availability

The original contributions presented in the study are included in the article/**Supplementary Material**, further inquiries can be directed to the corresponding author.

## Footnotes

1
https://arbeidoghelse.no/tema/arbeidsrettet-rehabilitering/

2
https://osha.europa.eu/sites/default/files/Work_related_MSDs_prevalence_costs_and_demographics_in_EU_summary.pdf

3
https://www.who.int/standards/classifications/international-classification-of-functioning-disability-and-health

4
https://cdn.who.int/media/docs/default-source/classification/icf/icfbeginnersguide.pdf?sfvrsn=eead63d3_4&download=true

5
https://www.psychiatry.org/File%20Library/Psychiatrists/Practice/DSM/APA_DSM5_Cultural-Formulation-Interview.pdf

6
https://arbeidoghelse.no/wp-content/uploads/2023/01/Arbeidsrettet-rehabilitering-kort-fortalt-revidert-13-01-2023.pdf

7
https://www.muskelskjeletthelse.no/om-radet-for-muskelskjeletthelse/

8
https://arbeidoghelse.no/fagveileder/om-arr/arr-i-spesialisthelsetjenesten/

9
https://arbeidoghelse.no/arbeid-og-helse-den-nye-strategien-er-klar/

10
https://www.nav.no/hva-er-nav

## References

[1] Bevan S . Economic impact of musculoskeletal disorders (Msds) on work in Europe. Best Pract Res Clin Rheumatol. (2015) 29:356–73. doi: 10.1016/j.berh.2015.08.002, PMID: 26612235

[2] Briggs AM Woolf AD Dreinhöfer K Homb N Hoy DG Kopansky-Giles D . Reducing the global burden of musculoskeletal conditions. Bull World Health Organ. (2018) 96:366–8. doi: 10.2471/BLT.17.204891, PMID: 29875522 PMC5985424

[3] Nasjonalt kompetansesenter for arbeidsrettet rehabilitering . Norwegian national competence center for occupational rehabilitation. Rauland: Arbeidsrettet Rehabilitering [Occupational Rehabilitation] ARBEIDOGHELSE.NO (2024). Available online at: https://arbeidoghelse.no/tema/arbeidsrettet-rehabilitering/ (Accessed October 7, 2024).

[4] de Kok J Vroonhof P Snijders J Roullis K Clarke M Peereboom K . Executive Summary: European Agency for Safety and Health at Work. Luxembourg (2019). Available online at: https://osha.europa.eu/sites/default/files/Work_related_MSDs_prevalence_costs_and_demographics_in_EU_summary.pdf (Accessed November 20, 2024).

[5] Rodrigues EV Gomes ARS Tanhoffer AIP Leite N . Effects of exercise on pain of musculoskeletal disorders: A systematic review. Acta Ortop Bras. (2014) 22:334–8. doi: 10.1590/1413-78522014220601004, PMID: 25538482 PMC4273961

[6] Robroek SJ Nieboer D Järvholm B Burdorf A . Educational differences in duration of working life and loss of paid employment. Scand J Work Environ Health. (2020) 46:77–84. doi: 10.5271/sjweh.3843, PMID: 31419303

[7] Sturgeon JA Zautra AJ . Social pain and physical pain: shared paths to resilience. Pain Manag. (2016) 6:63–74. doi: 10.2217/pmt.15.56, PMID: 26678402 PMC4869967

[8] Stilwell P Harman K . An enactive approach to pain: beyond the biopsychosocial model. Phenomenol Cognit Sci. (2019) 18:637–65. doi: 10.1007/s11097-019-09624-7

[9] Mardian AS Hanson ER Villarroel L Karnik AD Sollenberger JG Okvat HA . Flipping the pain care model: A sociopsychobiological approach to high-value chronic pain care. Pain Med. (2020) 21:1168–80. doi: 10.1093/pm/pnz336, PMID: 31909793

[10] Cohen M Quintner J Van Rysewyk S . Reconsidering the international association for the study of pain definition of pain. Pain Rep. (2018) 3:e634. doi: 10.1097/PR9.0000000000000634, PMID: 29756084 PMC5902253

[11] The Lancet . Rethinking chronic pain. Lancet. (2021) 397:2023. doi: 10.1016/S0140-6736(21)01194-6, PMID: 34062132

[12] Cohen SP Vase L Hooten WM . Chronic pain: an update on burden, best practices, and new advances. Lancet. (2021) 397:2082–97. doi: 10.1016/S0140-6736(21)00393-7, PMID: 34062143

[13] Yun D Choi J . Person-centered rehabilitation care and outcomes: A systematic literature review. Int J Nurs Stud. (2019) 93:74–83. doi: 10.1016/j.ijnurstu.2019.02.012, PMID: 30870614

[14] Jesus TS Bright FA Pinho CS Papadimitriou C Kayes NM Cott CA . Scoping review of the person-centered literature in adult physical rehabilitation. Disabil Rehabil. (2019), 43:1626–36. doi: 10.1080/09638288.2019.1668483, PMID: 31553633

[15] Thórarinsdóttir K Kristjánsson K Gunnarsdóttir TJ Björnsdóttir K . Facilitation of a person-centered approach in health assessment of patients with chronic pain: an ethnographic study. Qual Health Res. (2019) 29:471–83. doi: 10.1177/1049732318770628, PMID: 29685099

[16] Engel GL . The need for a new medical model: A challenge for biomedicine. Science. (1977) 196:129–36. doi: 10.1126/science.84746 847460

[17] World Health Organization (WHO) . International classification of functioning, disability and health (Icf). Geneva: WHO (2024). Available online at: https://www.who.int/standards/classifications/international-classification-of-functioning-disability-and-health (Accessed January 5, 2024).

[18] Wade DT Halligan PW . The biopsychosocial model of illness: A model whose time has come. Clin Rehabil. (2017) 31:995–1004. doi: 10.1177/026921551770989, PMID: 28730890

[19] Meints S Edwards R . Evaluating psychosocial contributions to chronic pain outcomes. Prog Neuropsychopharmacol Biol Psychiatry. (2018) 87:168–82. doi: 10.1016/j.pnpbp.2018.01.017, PMID: 29408484 PMC6067990

[20] Mescouto K Olson RE Hodges PW Setchell J . A critical review of the biopsychosocial model of low back pain care: time for a new approach? Disabil Rehabil. (2022) 44:3270–84. doi: 10.1080/09638288.2020.1851783, PMID: 33284644

[21] Lima DD Alves VLP Turato ER . The phenomenological-existential comprehension of chronic pain: going beyond the standing healthcare models. Philos Ethics Humanit Med. (2014) 9:1–10. doi: 10.1186/1747-5341-9-2, PMID: 24410937 PMC3996192

[22] Böhmer MC la Cour P Schnell T . A randomized controlled trial of the sources of meaning card method: A new meaning-oriented approach predicts depression, anxiety, pain acceptance, and crisis of meaning in patients with chronic pain. Pain Med. (2021) 23:314–25. doi: 10.1093/pm/pnab321, PMID: 34730813

[23] Lilja A DeMarinis V Lehti A Forssén A . Experiences and explanations of mental ill health in a group of devout christians from the ethnic majority population in secular Sweden: A qualitative study. BMJ Open. (2016) 6. doi: 10.1136/bmjopen-2016-011647, PMID: 27797991 PMC5093464

[24] Carr DB Bradshaw YS . Time to flip the pain curriculum? Editorial views. Anesthesiology. (2014) 120:12–4. doi: 10.1097/ALN.0000000000000054, PMID: 24201031

[25] Flink IK Reme S Jacobsen HB Glombiewski J Vlaeyen JW Nicholas MK . Pain psychology in the 21st century: lessons learned and moving forward. SJPAIN. (2020) 20:229–38. doi: 10.1515/sjpain-2019-0180, PMID: 32242835

[26] World Health Organization (WHO) . Towards a common language for functioning, disability, and health. Icf Geneva: WHO (2002). Available online at: https://cdn.who.int/media/docs/default-source/classification/icf/icfbeginnersguide.pdf?sfvrsn=eead63d3_4&download=true (Accessed January 8, 2024).

[27] Sharma S Ferreira-Valente A de C Williams AC Abbott JH Pais-Ribeiro J Jensen MP . Group differences between countries and between languages in pain-related beliefs, coping, and catastrophizing in chronic pain: A systematic review. Pain Med. (2020) 21:1847–62. doi: 10.1093/pm/pnz373, PMID: 32044980 PMC7553014

[28] Kleinman A . Patient and healers in the context of culture. In: An exploration of the borderland between anthropology, medicine, and psychiatry. University of California press, Berkeley (1980).

[29] Foreword KA . DSM-5 Handbook on the Cultural Formulation Interview. Lewis-Fernández R Aggarwal NK Hinton L Hinton DE Kirmayer LJ , editors. Washington, DC: American Psychiatric Publishing (2016) p. xvii–xix.

[30] Lewis-Fernández R Aggarwal NK Kirmayer LJ . Introduction. In: Lewis-Fernández R Aggarwal NK Hinton L Hinton DE Kirmayer LJ , editors. DSM-5 Handbook on the Cultural Formulation Interview. Washington DC: American Psychiatric Publishing (2016). p. xxvii–xiv.

[31] American Psychiatric Association . Diagnostic and statistical manual of mental disorders, fifth edition, text revision, DSM-5-tr. Washington, DC: American Psychiatric Association (2022).

[32] Regjeringen [Norwegian Government] . Statsbudsjettet 2026: Vil Styrke Budsjettet Med Nesten 600 Millionar Til Tiltak for Å Få Fleire I Jobb [State Budget 2026: Will Strengthen the Budget with Nearly 600 Million Nok for Initiatives to Get More People into Work]. Oslo: Regjeringen [Norwegian Government] (2025). Available online at: https://www.regjeringen.no/no/aktuelt/vil-styrke-budsjettet-med-nesten-600-millionar-til-tiltak-for-a-fa-fleire-i-jobb/id3124363/ (Accessed October 10, 2025).

[33] Ohm E Madsen C Gravseth HM Brage S Grøholt EK Alver K . Post-injury long-term sickness absence and risk of disability pension: the role of socioeconomic status. Injury. (2024) 55:111480. doi: 10.1016/j.injury.2024.111480, PMID: 38452702

[34] DeMarinis V . An existential healthcare framework for investigating pastoral care and the pastoral caring process in a postmodern context. In: Pastoral care, existential health, and existential epidemiology a swedish postmodern case study. Verbum, Stockholm (2003). p. 44–6.

[35] Haug SHK DeMarinis V Danbolt LJ Kvigne K . The illness reframing process in an ethnic-majority population of older people with incurable cancer: variations of cultural- and existential meaning-making adjustments. Ment Health Relig Cult. (2016) 19:150–63. doi: 10.1080/13674676.2015.1126705

[36] Vattø IE Haug SH DeMarinis V Lien L Danbolt LJ . The significance ascribed to contacting a diaconal suicide-prevention crisis line in Norway: A qualitative study. Ment Health Relig Cult. (2020) 23:113–26. doi: 10.1080/13674676.2020.1763281

[37] Lloyd C Klinteberg B DeMarinis V . Emotion regulation and existential meaning-making in young women with mental ill-health concerns–a qualitative study. JPBS. (2016) 1:001–010. doi: 10.19080/PBSIJ.2016.01.555553

[38] Haug SHK Danbolt LJ Kvigne K Demarinis V . Older people with incurable cancer: existential meaning-making from a life-span perspective. Palliat Support Care. (2016) 14:20–32. doi: 10.1017/S1478951515000644, PMID: 26062404

[39] Haug SHK Danbolt LJ Kvigne K DeMarinis V . How older people with incurable cancer experience daily living: A qualitative study from Norway. Palliat Support Care. (2014) 19:1037–48. doi: 10.1017/S1478951514001011, PMID: 25159499

[40] DeMarinis V . The impact of post-modernization on existential health in Sweden: psychology of religion’s function in existential public health analysis. Arch Psychol Relig. (2008) 30:57–74. doi: 10.1163/157361208X316962

[41] DeMarinis V Ulland D Karlsen KE . Philosophy’s role for guiding theory and practice in clinical contexts grounded in a cultural psychiatry focus: A case study illustration from southern Norway. WCPRR. (2011) 6:47–56. Availale online at: https://www.worldculturalpsychiatry.org/wp-content/uploads/2019/08/09-Philosophy-V06N1.pdf.

[42] Ulland D DeMarinis V . Understanding and working with existential information in a norwegian adolescent psychatry context: A need and a challenge. Ment Health Relig Cult. (2014) 17:582–93. doi: 10.1080/13674676.2013.871241

[43] American Psychiatric Association . Cultural Formulation Interview. American Psychiatric Association (2013). Available online at: https://www.psychiatry.org/File%20Library/Psychiatrists/Practice/DSM/APA_DSM5_Cultural-Formulation-Interview.pdf (Accessed October 7, 2024).

[44] Lewis-Fernandez R Aggarwal NK Kirmayer LJ . Cultural formulation before DSM-5. In: Lewis-Fernandez R Aggarwal NK Hinton L Hinton DE Kirmayer LJ , editors. DSM-5 Handbook on the Cultural Formulation Interview. Washington, DC: American Psychiatric Publishing (2016). p. 1–26.

[45] Aggarwal NK Jiménez-Solomon O Lam PC Hinton L Lewis-Fernández R . The core and informant Cultural Formulation Interviews in DSM-5. In: Lewis-Fernández R Aggarwal NK Hinton L Hinton DE Kirmayer LJ , editors. DSM-5 Handbook on the Cultural Formulation Interview. Washington DC: American Psychiatric Publishing (2016). p. 27–44.

[46] Aggarwal NK Cedeno K Lewis-Fernandez R . Patient and clinician communication practices during the DSM-5 cultural formulation interview field trial. Anthropol Med. (2020) 27:192–211. doi: 10.1080/13648470.2019.1641014, PMID: 31550913 PMC7093248

[47] Lewis-Fernandez R Aggarwal NK Lam PC Galfalvy H Weiss MG Kirmayer LJ . Feasibility, acceptability and clinical utility of the Cultural Formulation Interview: mixed-methods results from the DSM-5 international field trial. Br J Psychiatry. (2017) 210:290–7. doi: 10.1192/bjp.bp.116.193862, PMID: 28104738

[48] Aggarwal NK Desilva R Nicasio AV Boiler M Lewis-Fernandez R . Does the Cultural Formulation Interview for the fifth revision of the diagnostic and statistical manual of mental disorders (DSM-5) affect medical communication? A qualitative exploratory study from the new york site. Ethn Health. (2015) 20:1–28. doi: 10.1080/13557858.2013.85772, PMID: 25372242 PMC4221811

[49] Jones-Lavallee A Bernard G Taing J Leanza Y . The state of current knowledge on the Cultural Formulation Interview: A scoping review. J Psychopathol Behav Assess. (2023) 45:265–76. doi: 10.1007/s10862-022-10009-5

[50] Jarvis GE Kirmayer LJ Gómez-Carrillo A Aggarwal NK Lewis-Fernández R . Update on the Cultural Formulation Interview. Focus. (2020) 18:40–6. doi: 10.1176/appi.focus.20190037, PMID: 32047396 PMC7011218

[51] Wallin MI DeMarinis V Nevonen L Bäärnhielm S . What information did the DSM-5 Cultural Formulation Interview provide when used with swedish-speaking patients in a psychiatric setting in stockholm? Front Psychiatry. (2024) 15:1377006. doi: 10.3389/fpsyt.2024.1377006, PMID: 38840947 PMC11151123

[52] Svamo NTØ Haug SHK DeMarinis V . I need to get back to a normal life”: using the core DSM-5 Cultural Formulation Interview to explore existential themes shared by adolescents in specialized mental healthcare in Norway. Front Psychol. (2025) 16:3389/fpsyg.2025.1652189. doi: 10.3389/fpsyg.2025.1652189, PMID: 41312262 PMC12646933

[53] Ortega RC Garner WE . Using DSM-5 and icf tools to understand client cultural and environmental perspectives. JARC. (2016) 47:27–33. doi: 10.1891/0047-2220.47.2.27

[54] Peterson DB . Critical issues for mental health management in vocational rehabilitation: DSM-5, ICD-10- cm, and implementing the whodas. In: Escorpizo R Brage S Homa D Stucki G Brage S , editors. Handbook of vocational rehabilitation and disability evaluation: application and implementation of the icf, edited by reuben escorpizo. Publishing Switzerland: Springer International Publishing AG (2014). p. 317–33.

[55] Helsedirektoratet [Norwegian Directorate of health] . Lovgrunnlaget for spesialisthelsetjenestens ansvar for habilitering og rehabilitering [the legal basis for the specialist health service’s responsibility for habilitation and Rehabilitation]. Oslo: Norwegian Directorate of Health (2023).

[56] Nasjonalt kompetansesenter for arbeidsrettet rehabilitering [Norwegian national competence center for occupational rehabilitation] . Arbeidsrettet Rehabilitering- Kort Fortalt [Occupational Rehabilitation- Short Description]: ARBEIDOGHELSE.NO (2024). Available online at: https://arbeidoghelse.no/wp-content/uploads/2023/01/Arbeidsrettet-rehabilitering-kort-fortalt-revidert-13-01-2023.pdf (Accessed October 7, 2024).

[57] Gjesdal S Holmaas TH Monstad K Hetlevik Ø . New episodes of musculoskeletal conditions among employed people in Norway, sickness certification and return to work: A multiregister-based cohort study from primary care. BMJ Open. (2018) 8:e017543. doi: 10.1136/bmjopen-2017-017543, PMID: 29540405 PMC5857691

[58] Rådet for muskel - og skjeletthelse [Council for Musculoskeletal Health] . Om Oss [About Us]: Rådet for muskel- og skjeletthelse (2023). Available online at: https://www.muskelskjeletthelse.no/om-radet-for-muskelskjeletthelse/ (Accessed November 27, 2023).

[59] Nasjonalt kompetansesenter for arbeidsrettet rehabilitering [Norwegian national competence center for occupational rehabilitation] . Arbeidsrettet Rehabilitering I Spesialisthelsetjenesten [Occupational Rehabilitation in Specialized Health Care]: ARBEIDOGHELSE.NO (2024). Available online at: https://arbeidoghelse.no/fagveileder/om-arr/arr-i-spesialisthelsetjenesten/ (Accessed October 7, 2024).

[60] NAV Arbeids - og Velferdsetaten og Helsedirektoratet [Norwegian Labor and Welfare Administration and Norwegian Directorate of health] . Strategi for Fagfeltet Arbeid Og Helse [Strategy for the Field of Work and Health]: ARBEIDOGHELSE.NO (2021). Available online at: https://arbeidoghelse.no/arbeid-og-helse-den-nye-strategien-er-klar/ (Accessed November 20, 2024).

[61] (2011). Forskrift Om Habilitering, Rehabilitering Og Koordinator [Regulations on Habilitation, Rehabilitation and Coordinator], 2024.

[62] Regjeringen [Norwegian Government] . Nou; Tid for Handling — Personellet I En Bærekraftig Helse- Og Omsorgstjeneste [White Paper; Time for Action- the Personnel in Sustainable Health and Care Services]. Oslo: Departementenes sikkerhets- og serviceorganisasjon Tr (2023). NOU: 2023-4: Regjeringen.

[63] Aggarwal NK Glass A Tirado A Boiler M Nicasio A Alegria M . The development of the DSM-5 Cultural Formulation Interview-fidelity instrument (Cfi-fi): A pilot study. JHCPU. (2014) 25:1397–417. doi: 10.1353/hpu.2014.0132, PMID: 25130248 PMC4306341

[64] Elo S Kyngäs H . The qualitative content analysis process. J Adv Nurs. (2008) 62:107–15. doi: 10.1111/j.1365-2648.2007.04569.x, PMID: 18352969

[65] Nav Arbeids og velferdsetaten . Norwegian Labor and Welfare Administration]. Kva Er Nav? [What Is Nav]? Nav Arbeids og velferdsetaten (2024). Available online at: https://www.nav.no/hva-er-nav (Accessed November 26, 2024).

[66] Aggarwal NK Lam P Castillo EG Weiss MG Diaz E Alarcon RD . How do clinicians prefer cultural competence training? Findings from the DSM-5 Cultural Formulation Interview field trial. Acad Psychiatry. (2016) 40:584–91. doi: 10.1007/s40596-015-0429-3, PMID: 26449983 PMC4826320

[67] Wallin MI Dahlin M Nevonen L Bäärnhielm S . Patients’ and clinicians’ Experiences of the DSM-5 Cultural Formulation Interview: A mixed method study in a swedish outpatient setting. Transcult Psychiatry. (2020). 57:542–55. doi: 10.1177/1363461520938917, PMID: 32646300 PMC7488836

[68] Muralidharan A Schaffner RM Hack S Jahn DR Peeples AD Lucksted A . I got to voice what’s in my heart”: participation in the Cultural Formulation Interview—Perspectives of consumers with psychotic disorders. J Psychosocial Rehabil Ment Health. (2017) 4:35–43. doi: 10.1007/s40737-017-0076-y

[69] Lewis-Fernandez R Aggarwal NK Kirmayer LJ . The Cultural Formulation Interview: progress to date and future directions. Transcult Psychiatry. (2020) 57:487–96. doi: 10.1177/1363461520938273, PMID: 32838656

[70] Morgan S Yoder LH . A concept analysis of person-centered care. J holistic Nurs. (2012) 30:6–15. doi: 10.1177/0898010111412189, PMID: 21772048

