# Supplementary material for: Efficacy testing of the DSM-5 Cultural Formulation Interview for patients in vocational rehabilitation in Norway
Source: Front Psychiatry. 2026 Feb 23;16:1546150. doi: 10.3389/fpsyt.2025.1546150 (PMC12968993; doi:10.3389/fpsyt.2025.1546150)
Supplement: Supplementary file 1 [file DataSheet1.pdf]

Supplementary material 1, DSM-5 core Cultural Formulation Interview.
Adapted version for vocational rehabilitation, Norway.

Sigrid Helene Kjørven Haug, Research Center for Existential Health, Innlandet Hospital Trust, Ottestad, Norway; Faculty of Social and Health Sciences, University of Inland, Elverum, Norway
Valerie DeMarinis, Research Center for Existential Health, Innlandet Hospital Trust, Ottestad, Norway; Department of Public Health and Clinical Medicine, Umeå University, Sweden

Adaptations are marked in bold text.

INTERVIEW GUIDE	INSTRUCTIONS TO THE INTERVIEWER ARE ITALICIZED
The following questions aim to clarify key aspects of the presenting clinical problem from the point of view of the individual and other members of the individual's social network (i.e., family, friends, or others involved in current problem). This includes the problem's meaning, potential sources of help, and expectations for services.	INTRODUCTION FOR THE INDIVIDUAL:<br>I would like to understand the problems that bring you here so that I can help you more effectively. I want to know about your experience and ideas. I will ask some questions about what is going on and how you are dealing with it. Please remember there are no right or wrong answers.
**CULTURAL DEFINITION OF THE PROBLEM**<br>Explanatory Model, Level of Functioning)	
Elicit the individual's view of core problems and key concerns. Focus on the individual's own way of understanding the problem. Use the term, expression, or brief description elicited in question 1 to identify the problem in subsequent questions (e.g., "your conflict with your son").<br><br>Ask how individual frames the problem for members of the social network.<br><br>Focus on the aspects of the problem that matter most to the individual	1.What brings you here today? IF INDIVIDUAL GIVES FEW DETAILS OR ONLY MENTIONS SYMPTOMS OR A MEDICAL DIAGNOSIS, PROBE: People often understand their problems in their own way, which may be similar to or different from how doctors describe the problem. How would you describe your problem?<br>2. Sometimes people have different ways of describing their problem to their family, friends, or others in their community. How would you describe your problem to them?<br>3. What troubles you most about your problem **physically and mentally? What are the biggest consequences of these struggles for your daily life?**
**CULTURAL PERCEPTIONS OF CAUSE, CONTEXT, AND SUPPORT**<br>CAUSES<br>(Explanatory Model, Social Network, Older Adults)	
This question indicates the meaning of the condition for the individual, which may be relevant for clinical care.<br>Note that individuals may identify multiple causes, depending on the facet of the problem they are considering	4. Why do you think this is happening to you? What do you think are the causes of your [PROBLEM]?<br>PROMPT FURTHER IF REQUIRED: Some people may explain their problem as the result of bad things that happen in their life, problems

Focus on the views of members of the individual's social network. These may be diverse and vary from the individual's	with others, a physical illness, a spiritual reason, or many other causes<br>5. What do others in your family, your friends, or others in your community think is causing your [PROBLEM]?
STRESSORS and SUPPORT<br>(Social Network, Caregivers, Psychosocial Stressors, Religion and Spirituality, Immigrants and Refugees, Cultural Identity, Older Adults, Coping and Help Seeking)	
Elicit information on the individual's life context, focusing on resources, social supports, and resilience. May also probe other supports (e.g., from co-workers, from participation in religion or spirituality).<br><br>Focus on stressful aspects of the individual's environment. Can also probe, e.g., relationship problems, difficulties at work or school, or discrimination.	6. Are there any kinds of support that make your [PROBLEM] better, such as support from family, friends, or others?<br>**Are there any kinds of support that make your [PROBLEM] better in relation to your work? Are there any kinds of support that help you in dealing with your gradual loss of work capability?**<br>7. Are there any kinds of stresses that make your [PROBLEM] worse, such as difficulties with money, or family problems?<br>**Are there any kinds of stress that make your [PROBLEM] worse in relation to your work? How have you experienced the stress connected to your gradual loss of work capability? If you have had contact with NAV, how have you experienced it?**
ROLE OF CULTURAL IDENTITY<br>(Cultural Identity, Psychosocial Stressors, Religion and Spirituality, Immigrants and Refugees, Older Adults, Children and Adolescents)	
Ask the individual to reflect on the most salient elements of his or her cultural identity. Use this information to tailor questions 9–10 as needed.<br>Elicit aspects of identity that make the problem better or worse. Probe as needed (e.g., clinical worsening as a result of discrimination due to migration status, race/ethnicity, or sexual orientation).<br>Probe as needed (e.g., migration-related problems; conflict across generations or due to gender roles)	Sometimes, aspects of people's background or identity can make their [PROBLEM] better or worse. By background or identity, I mean, for example, the communities you belong to, the languages you speak, where you or your family are from, your race or ethnic background, your gender or sexual orientation, or your faith or religion.<br>8. For you, what are the most important aspects of your background or identity?<br>**What do you perceive as your resources? What do you perceive as your problems?**<br><br>9. Are there any aspects of your background or identity that make a difference to your [PROBLEM]?<br>10. Are there any aspects of your background or identity that are causing other concerns or difficulties for you?
**CULTURAL FACTORS AFFECTING SELF-COPING AND PAST HELP SEEKING**	

SELF-COPING<br>(Coping and Help Seeking, Religion and Spirituality, Older Adults, Caregivers, Psychosocial Stressors)	
Clarify self-coping for the problem.	11. Sometimes people have various ways of dealing with problems like [PROBLEM]. What have you done on your own to cope with your [PROBLEM]?<br>**What have you done yourself to manage your pain?**<br>**What have you done yourself to manage your everyday life?**<br>**What gives you strength and courage to move on?**<br>**What have you done on your own to cope with your work or your employment situation?**
PAST HELP SEEKING<br>(Coping and Help Seeking, Religion and Spirituality, Older Adults, Caregivers, Psychosocial Stressors, Immigrants and Refugees, Social Network, Clinician-Patient Relationship)	
Elicit various sources of help (e.g., medical care, mental health treatment, support groups, work-based counseling, folk healing, religious or spiritual counseling, other forms of traditional or alternative healing).<br>Probe as needed (e.g., “What other sources of help have you used?”).<br>Clarify the individual’s experience and regard for previous help	12. Often, people look for help from many different sources, including different kinds of doctors, helpers, or healers. In the past, what kinds of treatment, help, advice, or healing have you sought for your [PROBLEM]?<br>PROBE IF DOES NOT DESCRIBE USEFULNESS OF HELP RECEIVED:<br>What types of help or treatment were most useful? Not useful?
BARRIERS<br>(Coping and Help Seeking, Religion and Spirituality, Older Adults, Psychosocial Stressors, Immigrants and Refugees, Social Network, Clinician-Patient Relationship)	
Clarify the role of social barriers to help seeking, access to care, and problems engaging in previous treatment. Probe details as needed (e.g., “What got in the way?”).	13. Has anything prevented you from getting the help you need? PROBE AS NEEDED: For example, money, work or family commitments, stigma or discrimination, or lack of services that understand your language or background?
**CULTURAL FACTORS AFFECTING CURRENT HELP SEEKING**<br>PREFERENCES<br>(Social Network, Caregivers, Religion and Spirituality, Older Adults, Coping and Help Seeking)	
Clarify individual’s current perceived needs and expectations of help, broadly defined.<br>Probe if individual lists only one source of help (e.g., “What other kinds of help would be useful to you at this time?”).<br>Focus on the views of the social network regarding help seeking.	Now let’s talk some more about the help you need.<br>14. What kinds of help do you think would be most useful to you at this time for your [PROBLEM]?

	15. Are there other kinds of help that your family, friends, or other people have suggested would be helpful for you now?
CLINICIAN-PATIENT RELATIONSHIP<br>(Clinician-Patient Relationship, Older Adults)	
Elicit possible concerns about the clinic or the clinician-patient relationship, including perceived racism, language barriers, or cultural differences that may undermine goodwill, communication, or care delivery.<br>Probe details as needed (e.g., “In what way?”).<br>Address possible barriers to care or concerns about the clinic and the clinician-patient relationship raised previously.	Sometimes doctors and patients misunderstand each other because they come from different backgrounds or have different expectations.<br><br>16. Have you been concerned about this and is there anything that we can do to provide you with the care you need?
