# Supplementary material for: Efficacy testing of the DSM-5 Cultural Formulation Interview for patients in vocational rehabilitation in Norway
Source: Front Psychiatry. 2026 Feb 23;16:1546150. doi: 10.3389/fpsyt.2025.1546150 (PMC12968993; doi:10.3389/fpsyt.2025.1546150)
Supplement: Supplementary file 2 [file DataSheet2.docx]

**Supplementary material 2, T2 and T3 semi-structured interviews for patients**

The T2 interview is inspired by the Semi-structured, Debriefing Interview Questions on Feasibility, Acceptability, and Clinical Utility by Aggarwal and colleagues (2015). The questions have been modified for clinical populations in Norway by the second author (VDM). The T3 questions were developed by the first and second authors.

T2 interviews for patients:

1. Overall, how did you feel answering these questions about your perspective?

2. How did the questions affect what you talked about with the clinician?

3. How did the CFI affect your relationship with the clinician?

4. How different were these questions from those of your other clinicians?

5. How did the CFI affect what you think or feel about mental health care?

6. What was most helpful about the questions of the CFI? Least helpful?

7. Are there any particular CFI questions that you think should be changed or removed, perhaps because they were unclear? Are there any additional questions that were not asked during the CFI, but should be included?

8. How do you think the CFI might affect your care?

T3 interviews for patients:

1. What is your experience of being in this inpatient unit? What are you satisfied with and what could be better?

2. Have you given any thought to the timing of the CFI, could it have come later in the treatment period?

3. What have you found most important during your stay here?

(Topics from the CFI interview can be included here if relevant)

4. How have you found communication with the people who work here?

(Topics from the CFI can also be included here if relevant)

5. Have you found that the team has used information from the CFI in the treatment? In what ways?

6. Have your opinions and experiences been taken into account? In what ways?

7. How do you think your life will be after this treatment period?

Aggarwal, N. K., DeSilva, R., Nicasio, A. V., Boiler, M., & Lewis-Fernández, R. (2015). Does the Cultural Formulation Interview for the fifth revision of the diagnostic and statistical manual of mental disorders (DSM-5) affect medical communication? A qualitative exploratory study from the New York site. *Ethnicity and Health, 20*(1), 1-28.
