# Supplementary material for: Efficacy testing of the DSM-5 Cultural Formulation Interview for patients in vocational rehabilitation in Norway
Source: Front Psychiatry. 2026 Feb 23;16:1546150. doi: 10.3389/fpsyt.2025.1546150 (PMC12968993; doi:10.3389/fpsyt.2025.1546150)
Supplement: Supplementary file 3 [file DataSheet3.docx]

**Supplementary material 4**, **Cultural Formulation Interview – Fidelity Instrument (CFI-FI),** Aggarwal et al (2014)

| Item* | Mean | Range |
| --- | --- | --- |
| *Empathy: Did the clinician paraphrase or name the patient’s emotional state?* | 7.8 | 7-9 |
| *Patient centeredness: Did the clinician maintain a non-judgmental attitude (not arguing, confronting, or correcting the patient)?* | 9.7 | 9-10 |
| *Clarification: Did the clinician ask follow-up questions to understand unclear patient responses?* | 6.8 | 6-9 |
| *Word matching: Did the clinician use the patient’s preferred illness term whenever the CFI question stem included the term “[PROBLEM]”?* | 6.5 | 5-9 |
| *Illness narration: Did the clinician’s interactions help the patient construct and explore a narrative account of illness or did the clinician seem to rush through the CFI?* | 9.0 | 6-10 |
| *Drift: Did the clinician ask about topics during the CFI session that typically belong to the standard clinical interview (history of present illness, current medications, detailed psychiatric or medical history, family history, social history, mini-mental status examination)?* | 10 | 10 |
| *Order: Did the clinician ask about all topics in order as reflected in the CFI clinician guidelines?* | 8.3 | 8-10 |

Mean score of all 7 items 8.3
